# Intellectual disability-associated UNC80 mutations reveal inter-subunit interaction and dendritic function of the NALCN channel complex

**DOI:** 10.1038/s41467-020-17105-8

**Published:** 2020-07-03

**Authors:** Jinhong Wie, Apoorva Bharthur, Morgan Wolfgang, Vinodh Narayanan, Keri Ramsey, C4RCD Research Group, Newell Belnap, Ana Claasen, Amanda Courtright, Matt de Both, Matthew Huentelman, Sampathkumar Rangasamy, Ryan Richholt, Isabelle Schrauwen, Ashley L. Siniard, Szabolics Szelinger, Kimberly Aranda, Qi Zhang, Yandong Zhou, Dejian Ren

**Affiliations:** 1 Department of Biology, University of Pennsylvania, Philadelphia, PA 19104 USA; 2 Center for Rare Childhood Disorders, Translational Genomics Research Institute, Phoenix, AZ 85012 USA; 3 Department of Cellular and Molecular Physiology, Pennsylvania State University College of Medicine, Hershey, PA 17033 USA

**Keywords:** Ion channels in the nervous system, Physiology

## Abstract

The sodium-leak channel NALCN forms a subthreshold sodium conductance that controls the resting membrane potentials of neurons. The auxiliary subunits of the channel and their functions in mammals are largely unknown. In this study, we demonstrate that two large proteins UNC80 and UNC79 are subunits of the NALCN complex. UNC80 knockout mice are neonatal lethal. The C-terminus of UNC80 contains a domain that interacts with UNC79 and overcomes a soma-retention signal to achieve dendritic localization. UNC80 lacking this domain, as found in human patients, still supports whole-cell NALCN currents but lacks dendritic localization. Our results establish the subunit composition of the NALCN complex, uncover the inter-subunit interaction domains, reveal the functional significance of regulation of dendritic membrane potential by the sodium-leak channel complex, and provide evidence supporting that genetic variations found in individuals with intellectual disability are the causes for the phenotype observed in patients.

## Abstract (web summary)

The sodium-leak channel NALCN controls the resting membrane potentials of neurons. Here, the authors identified two subunits of NALCN, UNC80 and UNC79. Domains in UNC80, which are mutated in individuals with intellectual disability, interact to achieve the dendritic localization of NALCN complex.

## Introduction

The resting membrane potentials (RMPs) of mammalian neurons vary significantly among cells from different brain regions and within the same region. In some neurons such as the spiny striatal neurons, they can be as polarized as approximately −90 mV, close to the equilibrium potential of K^+^ (*E*_K_), whereas in other neurons such as the spontaneously firing neurons dissociated from cerebellar nuclei and hypothalamus, they can be as depolarized as approximately −40 mV^1–3^. Similarly, RMPs recorded from the same region, such as the suprachiasmatic nucleus (SCN), span a large range between approximately −70 to −50 mV^4^. In addition, some bistable neurons can have two RPMs that are different by as much as 30 mV, depending on the history of synaptic input or time of the day (for a review)^5^.

A major mechanism to generate RMPs above *E*_K_ is via Na^+^ conductances. The resting Na^+^ permeability (*P*_Na_) is very small (a few percent of *P*_K_), but regulating the permeability ratio (*P*_Na_/*P*_K_) provides a powerful way to control RMPs^6^. The resting K^+^ counductances are formed by many K^+^ channels including the 15 K2P leak channels and several voltage-gated K^+^ channels (for reviews)^7,8^. The resting Na^+^ conductance is contributed by several types of ion channels. In many CNS neurons, TTX-sensitive, voltage-gated Na^+^ channels (Na_V_s) generate persistent and resurgent currents, particularly at more depolarized RMPs (for reviews)^9,10^. At more hyperpolarized membrane potentials (MPs), the hyperpolarization-activated cation channels (HCNs) are active in many neurons^11^. In most neurons, there is also a TTX-resistant, voltage-independent true Na^+^ leak conductance formed by the NALCN channel^1,3,12,13^. Consistent with NALCN’s major contribution, RMPs recorded from neurons with NALCN disrupted are hyperpolarized compared with wild-type (WT) by ~10–20 mV, and are little sensitive to changes in extracellular Na^+^ concentration, as found in hippocampal, retrotrapezoid nucleus, SCN, and spinal cord neurons^12,14–16^. In the midbrain dopaminergic neurons, NALCN contributes to the spontaneous activities, and is inhibited by the D2 dopamine receptor and GABA-B receptor^17^. NALCN is also activated by neuropeptides substance P (SP) and neurotensin. Its contribution to SP’s excitatory action is dominant despite the peptide’s extensively studied modulation of several K^+^ channels: neurons lacking NALCN are not excited by SP, as found in several types of neurons from hippocampi, VTA, RTN, the pre-Botzinger complex and spinal cord^15,16,18,19^. In addition, the channel is inhibited by extracellular Ca^2+^ through the Ca^2+^-sensing receptor and G-proteins. Decreases in extracellular [Ca^2+^] ([Ca^2+^]_e_) activates the channel^20^. In NALCN knockout (KO) mice, drop in [Ca^2+^]_e_ to sub-millimolar no longer excites neurons as in WT, suggesting that an increase in Na^+^ leak is a major mechanism by which lowering [Ca^2+^]_e_ leads to neuronal excitation^20,21^.

Mutant animals from various species have established NALCN as one of the most essential ion channels. NALCK KO mice have severe apnea and die within 24 h of birth^12^. In *C. elegans* and *Drosophila*, NALCN mutant animals have disrupted locomotion, abnormal circadian rhythms, and altered sensitivity to anesthetics^22–26^. In humans, NALCN deficiency is associated with severe neurological phenotypes including hypotonia, central apnea, inability to sit or stand, lack of speech development, absence of meaningful communication, and severe intellectual disability^27–33^.

NALCN belongs to the superfamily of 24 transmembrane-spanning ion channel proteins that contain four homologous repeats of six transmembrane-spanning segments (4 × 6TM)^21,34,35^. This superfamily also includes the family of ten voltage-gated Ca^2+^ channels (Ca_V_s) and the family of nine Na_V_s. The protein complexes of Ca_V_ and Na_V_ families have been extensively studied^38,36,37^. In contrast, the subunit composition of NALCN has not been determined. In *C. elegans* and *Drosophila*, *NALCN* orthologs (*Nca-1*, *Nca-2* in *C. elegans*, NA in *Drosophila*) genetically interact with several other genes including *UNC-79*, *UNC-80*, *NLF-1*, *UNC-7*, and *Synaptojanin*^22–25,39,40^. NLF-1 is localized in the endoplasmic reticulum where it facilitates trafficking NALCN to the plasma membrane^39^. In mouse brains, NALCN physically interacts with both UNC79 and UNC80^18,20^. However, it is not known whether UNC80 and UNC79 are simply two of many NALCN-interacting proteins or they are exclusively associated with NALCN and can be considered as bona fide auxiliary subunits.

Despite the large size of UNC80 (3326 aa in the mouse isoform, 3258 in humans) and its high degree of conservation among vertebrates (97% identity between mouse and human, 33% between mouse and *C. elegans*), there is no functional domain predicted or experimentally identified. The in vivo function of UNC80 in mammals is also not established as no KO mouse has been reported. We and others have recently found human individuals with variations along the open reading frame of *UNC80*, including truncations at the very N-terminal (e.g., R51*) and the C-terminal ends (e.g., L2586*) of the protein (Supplementary Fig. 1). Those individuals have hypotonia, impaired speech development, severe intellectual disability, and premature death^41–47^. The genetic variations were largely discovered using whole-exome sequencing (WES). As for any WES-based diagnosis, the individuals also have other detected genetic variations in exons and likely also have undetected intronic variations that cannot be convincingly excluded as causes of the phenotypes. As a result, the causal relationship between the *UNC80* variations/mutations and the severe diseases remains to be firmly established.

In this study, we generated targeted UNC80 mutations in the mice to test the relationships. UNC80 null, like those of NALCN and UNC79, have severe apnea and die shortly after birth. The severe phenotype provides the strongest evidence that the phenotypes in the UNC80 human patients are the results of the mutations detected with WES. We also used the mutant mice to reveal UNC80 domains important for inter-subunit interaction and dendritic localization.

## Results

### Targeted disruption of UNC80 leads to severe apnea and neonatal lethality

**Fig. 1:**
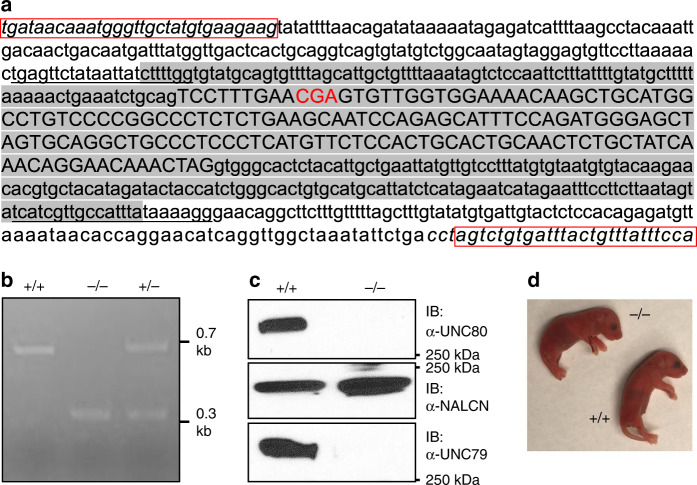
Targeted disruption of *UNC80* leads to apnea and neonatal lethality. **a** The design of *UNC80* knockout (KO) using the CRISPR technique to delete exon 3. Exon 3 sequence is in capital and the surrounding introns are in lower case. The 5′ and 3′ target sequences including the PAM motif (XGG) against which the two CRISPR sgRNAs targeted are underlined. Deleted sequences including exon 3 and part of the surrounding introns are shaded. Deletion of exon 3 (total of 157 nucleotides) leads to truncation after V47. The codon encoding R51 (CGA) corresponding to the residue mutated to a stop codon found in human patients are in red. PCR primers used for genotyping in **b** are in italic and boxed. **b** Genotyping PCR products from WT (+/+), heterozygote (+/−), and homozygous KO (−/−) pups using primers in **a**. **c** Total brain proteins from +/+ and *UNC80* −/− were blotted with anti-UNC80 (upper), anti-NALCN (middle), or anti-UNC79 (lower) antibody. **d** Representative appearances of WT and KO P0 pup. For (**b**) and (**c**), three or more independent repeats were performed with similar results. For apnea phenotype in the KO, see Supplementary Movie 1. Source data are provided as a Source Data file.

To test whether disruption in UNC80 is sufficient to lead to severe phenotypes in mammals, we used the CRISPR/Cas9 technique to generate a KO mouse line with UNC80 truncated at V47 (thereafter called UNC80 KO; Fig. 1a, b), close to R51, the position of a truncation found in several human patients [R51*, ^44^]. This truncation removes 3279 of 3326 residues of the protein (GenBank # NM_175510 as coordinate). As expected, an antibody raised against the C-terminal 15 residues failed to detect any UNC80 protein in whole brain lysate from mutant pups, confirming the lack of UNC80 protein expression (Fig. 1c).

Heterozygous *UNC80* KO mice were viable, fertile, and had no gross abnormality. From matings between heterozygous, pups were born with genotypes close to a Mendelian fashion (47 litters, 131^+/+^, 189^+/−^, and 85^−/−^). Homozygous mutant (−/−) pups appeared normal at birth (Fig. 1d). However, no −/− pups survived beyond 24 h of birth. Close inspection revealed that −/− pups had severe apnea (Supplementary Video 1). The apnea and neonatal lethality phenotypes are similar to those found in the *NALCN* KO^12^. In humans, the phenotypes found in individuals with *UNC80* null mutations and those with *NALCN* mutations are also similar to each other^27,28,44^. The severe phenotypes caused by the targeted disruption in UNC80 strongly support a causal relationship between the *UNC80* genetic variations and the phenotypes found in the human individuals.

### UNC80 is required for the NALCN-mediated TTX-resistant Na^+^ leak current and its regulation

**Fig. 2:**
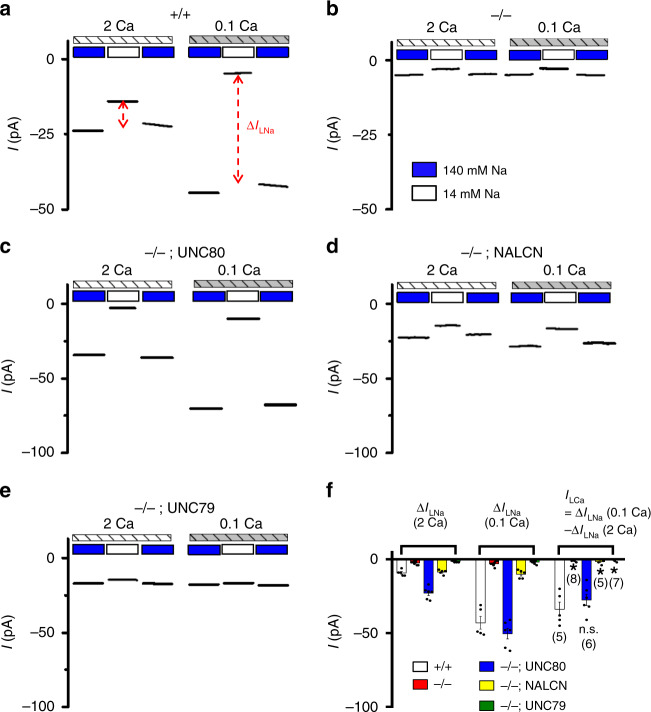
UNC80 is required for the TTX-resistant Na^+^ leak current and its regulation by extracellular Ca^2+^. **a**, **b** Representative TTX-resistant Na^+^ leak current recorded in hippocampal neurons cultured from WT (+/+) (**a**) and *UNC80* KO (−/−) (**b**) pups. Each trace represents 1 s of currents recorded at −70 mV in bath solutions with varying [Na^+^] (140 or 14 mM) and [Ca^2+^] (2 or 0.1 mM). The Na^+^-leak current (Δ*I*_LNa_) is calculated as the difference of current sizes between those recorded under 140 mM [Na^+^] and 14 mM [Na^+^], as indicated by dashed lines. **c**–**e** Similar to **a**, but recorded from *UNC80* KO neurons transfected with UNC80 (**c**), NALCN (**d**), or UNC79 (**e**) cDNA. **f** Averaged sizes of currents in **a**–**e**. *I*_LCa_ is the current activated by lowering [Ca^2+^] from 2 to 0.1 mM, as defined as the size difference of Δ*I*_LNa_ under the two [Ca^2+^] conditions). Data are presented as mean values ± SEM. Numbers of neurons are in parentheses. In (**f**), two sample *t* test of each group against the “+/+” group (*n* = 5) was performed: “−/−” (*n* = 8, *p* ≤ 0.001), “−/−; UNC80” (*n* = 6, *p* = 0.326), “−/−; NALCN” (*n* = 5, *p* ≤ 0.001), “−/−; UNC79” (*n* = 7, *p* ≤ 0.001). Asterisk “*” indicates *p* < 0.05. Source data are provided as a Source Data file.

Because of the similar phenotypes in *UNC80* and *NALCN* KOs and the association between the two proteins, we compared the NALCN-dependent Na^+^ leak current (Δ*I*_LNa_) in WT and *UNC80* KO neurons. Due to the small sizes of the whole-cell currents (~10 pA), we measured Δ*I*_LNa_ as the change in the sizes of holding current (at −70 mV) when bath [Na^+^] was lowered from 140 to 14 mM, in the presence of TTX and Cs^+^ to block Na_v_s and HCNs, respectively^3,12^ (Fig. 2a). The TTX-resistant Na^+^ leak current at subthreshold MPs is mediated by NALCN and is abolished in *NALCN* KO^12,14,15,19,48^.

Compared with WT, hippocampal neurons cultured from *UNC80* KOs had drastically decreased Δ*I*_LNa_ (WT: 12.0 ± 1.5 pA, *N* = 5; KO: 2.4 ± 0.4 pA, *N* = 8) (Fig. 2a, b, f). Transfecting an UNC80 cDNA driven by a strong CMV promoter into the KO neurons fully restored Δ*I*_LNa_, suggesting that the reduction of Δ*I*_LNa_ in the KO neurons was not a result of nonspecific developmental defect or off-target disruption of another gene by the CRISPR technique (Fig. 2c, f).

In mammalian neurons, a drop in extracellular Ca^2+^ concentration ([Ca^2+^]_e_), found under both physiological and pathophysiological conditions, generally excites neurons^49^. Lowering [Ca^2+^]_e_ to sub-millimolar activates an inward current^50–53^. We previously found that the current results from an increase in the NALCN-dependent current (*I*_NALCN_)^20^. Consistent with the obligate role of UNC80 in the regulation of *I*_NALCN_ by [Ca^2+^]_e_, lowering [Ca^2+^]_e_ from 2 to 0.1 mM increased Δ*I*_LNa_ in the WT by approximately threefold, but did not lead to obvious increase of Δ*I*_LNa_ in the KO neurons (Fig. 2b, f). Again, transfecting UNC80 cDNA fully rescued the Ca^2+^ sensitivity (Fig. 2c, f).

In total brain protein extracts, the level of NALCN protein in the KO was comparable to that in the WT (Fig. 1c). The drastically smaller Δ*I*_LNa_ in the KO neurons suggests that UNC80 potentiates *I*_NALCN_. Increasing the level of NALCN protein via NALCN cDNA transfection in the UNC80 KO neurons also increased Δ*I*_LNa_ (Fig. 2d, f), suggesting that NALCN can form channels in the absence of UNC80. However, Δ*I*_LNa_ without UNC80 was not potentiated by lowering [Ca^2+^]_e_, further confirming that *I*_NALCN_’s sensitivity to [Ca^2+^]_e_ requires UNC80.

In UNC80 KO brains, the UNC79 protein level was also drastically lower (Fig. 1c). We previously demonstrated that in UNC79 KO neurons, *I*_NALCN_ is insensitive to [Ca^2+^]_e_, presumably because of  a lowered UNC80 level^20^. Transfecting UNC79 cDNA into the *UNC79* KO neurons could rescue Δ*I*_LNa_’s [Ca^2+^]_e_ sensitivity^20^. In *UNC80* KO neurons, however, UNC79 transfection could not restore Δ*I*_LNa_, suggesting that the large reduction of Δ*I*_LNa_ in the *UNC80* KO was not due to the lack of UNC79 (Fig. 2e, f). These data further support the idea that UNC79 indirectly controls NALCN function through UNC80^21^.

### Haploinsufficient reduction in UNC80 function is associated with severe intellectual disability

In both humans and mice, individuals heterozygous for *UNC80* null mutations develop normally, are fertile and do not have obvious abnormalities such as lethality and severe intellectual disability, suggesting that a reduction in *UNC80* gene dosage by 50% is tolerated by the organisms. To determine the level of UNC80 function (measured as *I*_NALCN_) below which severe phenotypes are present in humans, we searched for *UNC80* mutations that retain residual function at a level below 50%.

**Fig. 3:**
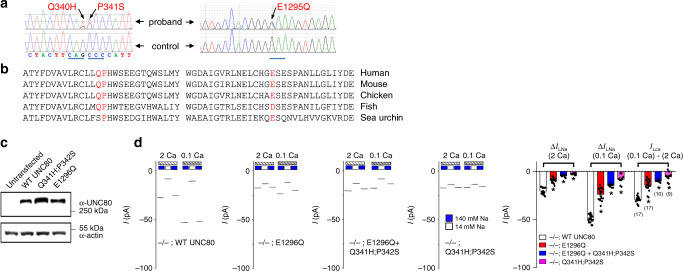
Haploinsufficient reduction in UNC80 is associated with severe intellectual disability. **a** Sanger sequencing chromatograms confirming the whole-exome sequence findings of genetic variations in the proband leading to protein changes of Q340H, P341S (left), and E1295Q (right). Upper: from the proband; lower: control. Codons encoding Q340 (CAG), P341 (CCC) and E1295 (GAA) are underlined. **b** Protein sequence alignments showing conservation in the Q340, P341 (left) and E1295 (right) regions. Accession numbers of the sequences used are NM_032504 (human), NP_780719 (mouse), XP_015144792 (chicken), XP_009300567 (zebrafish), and XP_011676014 (sea urchin). **c** Western blots with proteins prepared from non-transfected HEK293T cells and those transfected with mouse UNC80 wild type, Q341H;P342S (corresponding to human Q340H;P341S) or E1296Q (corresponding to human E1295Q) mutants, blotted with anti-UNC80 antibody (upper) or anti-actin (lower) for loading control. More than three independent repeats were performed with similar results. **d** TTX-resistant Na^+^ leak current recorded from cultured *UNC80* KO hippocampal neurons transfected with wild-type UNC80, E1296Q, Q341H;P342Q, or mixture of the two mutants, as indicated. Representative currents are in the left four subpanels and averaged current amplitudes are summarized in the right. Recordings were from −70 mV and were done with bath solutions with varying [Na^+^] (140 mM or 14 mM) and [Ca^2+^] (2 mM (2 Ca) or 0.1 mM (0.1 Ca)) (see Fig. 2 legend for details). Data are presented as mean values ± SEM. Numbers of neurons recorded are in parentheses. In the right bar graph, two sample *t* test of each group against the “−/−; WT UNC80” group (*n* = 17) was performed and the *p* values are as follows. Δ*I*_LNa_ (2 Ca): “−/−; E1296Q” (*n* = 17, *p* ≤ 0.001), “−/−; E1296Q + Q341H;P342S” (*n* = 10, *p* ≤ 0.001), “−/−; Q341H;P342S” (*n* = 9, *p* ≤ 0.001)). Δ*I*_LNa_ (0.1 Ca): “−/−; E1296Q” (*p* ≤ 0.001), “−/−; E1296Q + Q341H;P342S” (*p* ≤ 0.001), “−/−; Q341H;P342S” (*p* ≤ 0.001). Δ*I*_LCa_ (0.1 Ca–2 Ca): “−/−; E1296Q” (*p* ≤ 0.001), “−/−; E1296Q + Q341H;P342S” (*p* ≤ 0.001), “−/−; Q341H;P342S” (*p* ≤ 0.001). Asterisk “*” indicates *p* < 0.05. Source data are provided as a Source Data file.

One individual we came across has hypotonia, feeding difficulties, seizures, developmental delay, intellectual disability, and is nonverbal. His phenotype is similar to, although milder than, those found in the individuals with *UNC80* null mutations^44^. Using WES with samples from the individual and his parents, we detected biallelic variations in the *UNC80* gene (Fig. 3a). In one allele, he inherited variations of c.1020G>T and c.1021C>T (p.Q340_P341delinsHS) from his mother. In the other, he inherited c.3883G>C (p.E1295Q) from his father (Fig. 3a). The three residues (Q340, P341, and E1295) mutated in the individual are highly conserved among deuterostome animals, from sea urchins to fishes, and humans (Fig. 3b).

To determine whether the variations alter UNC80 function, we introduced into mouse UNC80 (97% identical to human UNC80) the corresponding mutations (Q341H;P342S for human p.Q340_P341delinsHS; E1296Q for human p.E1295Q). When transfected into HEK293T cells, the mutant cDNAs generated proteins at levels comparable to those of WT (Fig. 3c). When transfected into *UNC80* KO neurons, the Q341H;P342S mutant UNC80 generated little or no Δ*I*_LNa_ at 2 mM or 0.1 mM [Ca^2+^]_e_. Thus, the two-amino-acid variation found in one allele of the individual largely disrupts UNC80 function (Fig. 3d).

The E1296Q mutant partially restored Δ*I*_LNa_ when expressed in *UNC80* KO neurons. Like that from the WT-transfected neurons, the E1296Q -generated current was sensitive to [Ca^2+^]_e_. However, the sizes of the currents were only <50% of those from the WT cDNA-transfected ones under both 2 and 0.1 mM [Ca^2+^]_e_ (Fig. 3d).

In the individual carrying the essentially non-functional Q340H;P341S allele and the partially functional E1295Q allele, the UNC80-mediated *I*_NALCN_ is expected to be reduced by ~75%. Consistent with this prediction, co-transfection of both of the UNC80 mutant cDNA constructs at equal amount into *UNC80* KO neurons generated Δ*I*_LNa_ with a size of ~25% of those from WT UNC80-transfected ones (Fig. 3d). Taken together, these data suggest that while a reduction of 50% in *UNC80* gene dosage is tolerated, further reduction of UNC80 function to below 25% likely leads to severe phenotypes.

### UNC80 and UNC79 are subunits of the NALCN complex

**Fig. 4:**
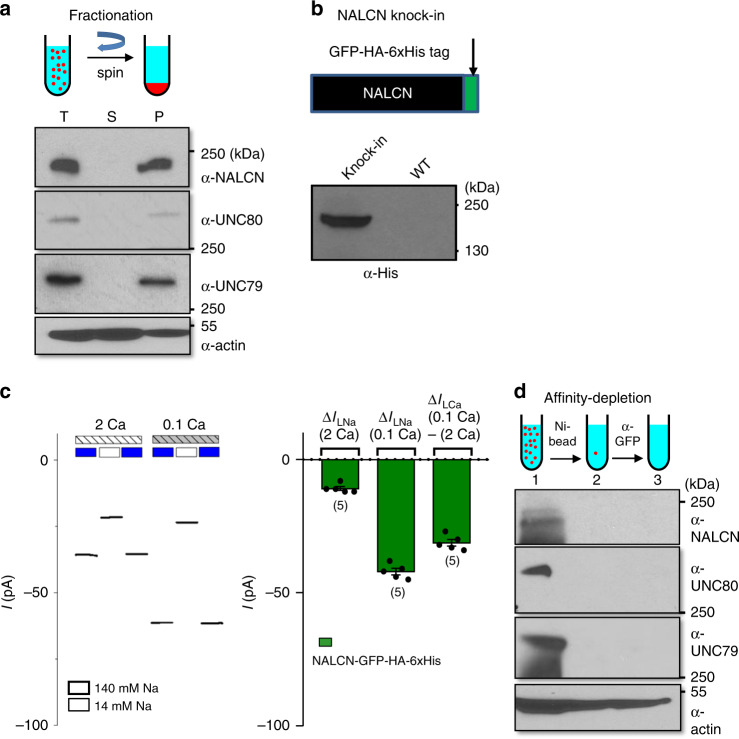
UNC80 and UNC79 are auxiliary subunits of the NALCN channel complex. **a** Protein fractionation demonstrating that UNC80 and UNC79 co-segregate with NALCN to the membrane fraction. Total proteins (T) from adult brains were centrifuged at 200,000 × *g* and separated into the cytosolic (supernatant, S) and microsomal membrane (pellet, P) fractions, as illustrated in the schematic diagram (upper). Each fraction was blotted with anti-NALCN, anti-UNC80, anti-UNC79, or anti-actin antibody. **b** A knock-in mouse line with NALCN tagged with GFP, HA, and His tags (NALCN-GFP-HA-His mice). Upper, schematic design. Lower, total brain proteins (100 μg) prepared from the triple-tagged mice and wild-type (non-tagged) mice were immunoblotted with anti-His antibody. **c** TTX-resistant Na^+^ leak currents recorded from neurons cultured from the KI pups (*n* = 5). Left, representative current traces. Right, averaged current amplitudes. Recordings were from −70 mV and were done with bath solutions with varying [Na^+^] (140 mM or 14 mM) and [Ca^2+^] (2 mM (2 Ca) or 0.1 mM (0.1 Ca)) (see Fig. 2 legend for details). Numbers of neurons recorded are in parentheses. **d** Protein depletion demonstrating that all UNC80 and UNC79 proteins are associated with NALCN. Total brain protein lysates were prepared from the NALCN-GFP-HA-His mice. NALCN was depleted by incubating with Ni column (binding to 6-His) followed by further immune depletion with anti-GFP antibody. Lysates before (lane 1) and after (lane 2: with Ni-beads, lane 3: with α-GFP agarose) depletion were blotted with anti-NALCN, anti-UNC79, or anti-UNC80 antibody. Anti-actin was used as a control. For (**a**, **b**, **d**), three or more independent repeats were performed with similar results. Data are presented as mean values ± SEM. Source data are provided as a Source Data file.

UNC80’s essential physiological roles could be primarily through its regulation of NALCN and/or through its other functions. We used protein fractionation and protein depletion assays to test whether UNC80 is exclusively associated with the NALCN complex. Consistent with being a 24 transmembrane-spanning protein, NALCN from mouse brains co-segregated with the microsomal fraction after centrifugation at >100,000 × *g* (Fig. 4a). UNC80 and UNC79 do not have obvious predictable transmembrane-spanning segments. Nevertheless, both of them co-segregated with NALCN in the microsomal fraction, suggesting that the two proteins are membrane associated (Fig. 4a). We further tested whether depleting NALCN from solubilized total brain protein also depletes UNC80 and UNC79. To facilitate the depletion, we generated a knock-in (KI) mouse line in which NALCN is fused with a GFP-HA-6xHis triple tag (the NALCN-GFP-HA-His mice, Fig. 4b), against which commercially antibodies and histidine-binding resins suitable for affinity depletion are readily available. Anti-His antibody detected tagged NALCN in protein prepared from the KI brains but not in that from WT (Fig. 4b). The sizes of the Na^+^-leak current and its Ca^2+^ sensitivity recorded from neurons cultured from the KI mice (Fig. 4c) are comparable to those of the WT (Fig. 2f). In addition, the KI mice are viable, fertile, and do not have gross abnormality, suggesting that the tagged NALCN functionally replaced the native one. After NALCN was depleted with histidine-binding Ni-column and anti-GFP antibody, both UNC80 and UNC79 became undetectable, suggesting that nearly all UNC80 and UNC79 proteins are in the NALCN complex (Fig. 4d). Based on this finding of apparently exclusive physical association of UNC80 and UNC79 with NALCN, and previous findings of functional and genetic interaction among the three, we propose that UNC80 and UNC79 are auxiliary subunits of the NALCN complex.

### UNC80’s N-terminal half interacts with NALCN

**Fig. 5:**
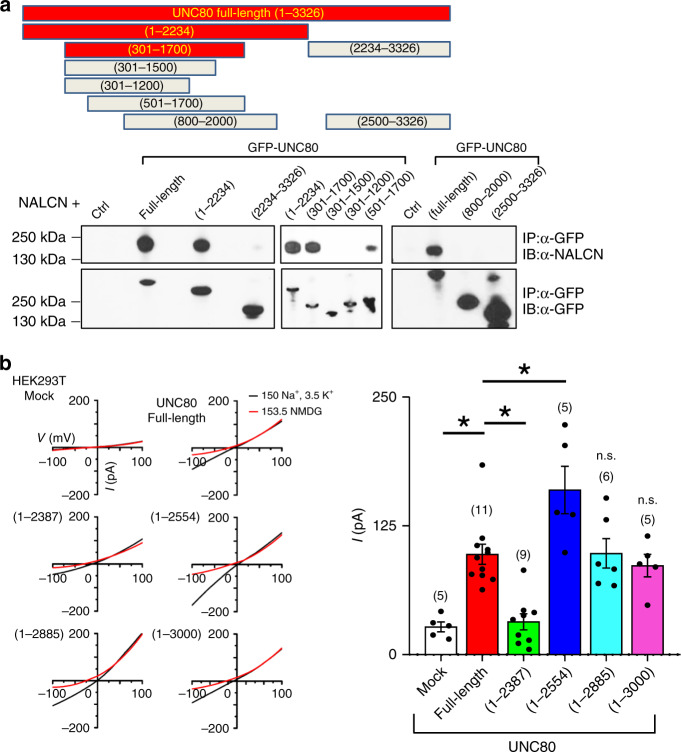
UNC80’s N-terminal half interacts with NALCN. **a** Co-immunoprecipitation assays to locate fragments on UNC80 required for its association with NALCN. Upper panels, schematic presentation of mouse UNC80 truncation mutants used in the studies. Lower panels, HEK293T cells were co-transfected with NALCN and GFP-tagged full-length or truncated UNC80 containing residues as indicated. Cell lysates were immunoprecipitated (IP) with anti-GFP followed by immunoblotting with anti-GFP (lower) or anti-NALCN (top). GFP was used in “ctrl”. It migrated at ~20 kDa (outside the molecular ranges shown) and is not visible in the blots. More than three independent repeats were performed with similar results. **b** *I*_NALCN_ from cells transfected with NALCN and wild-type or truncated UNC80 mutants as indicated. Recordings were done using a ramp protocol from −100 to +100 mV in 1 s (holding voltage *V*_h_ = 0 mV). To ensure that the current was not from nonspecific leak, bath cations were replaced with large non-permeant ion NMDG after each recording (see “Methods” for details). Bar graphs show averaged *I*_NALCN_ amplitude (at −100 mV). Two sample *t* test of each group against the “full-length” group (*n* = 11) was performed and the *p* values are: “Mock” (*n* = 5, *p* ≤ 0.001), “1–2387” (*n* = 9, *p* ≤ 0.001), “1–2554” (*n* = 5, *p* = 0.019), “1–2885” (*n* = 6, *p* = 0.818), “1–3000” (*n* = 5, *p* = 0.933). Asterisk “*” indicates *p* < 0.05. Data are presented as mean values ± SEM. Numbers of cells are in parentheses. Source data are provided as a Source Data file.

UNC80 associates with NALCN when the two are co-transfected into HEK293T cells^18^. We used co-immunoprecipitation assays to define the regions that mediate UNC80’s interaction with NALCN. The N-terminal half (aa 300–1700) was sufficient for the UNC80–NALCN association. Further deletion from the N- or C-terminal side greatly diminished the association (Fig. 5a).

We also used deletions to determine the regions functionally important for UNC80’s potentiation of *I*_NALCN_. When UNC80 and NALCN are co-transfected in HEK293T cells, cellular dialysis with a peptide Src activator via pipette perfusion potentiates *I*_NALCN_ in an UNC80-dependent fashion^18,42^ (Fig. 5b). UNC80 with residues deleted from the C-terminal end up to aa 2554 was fully functional in supporting *I*_NALCN_ (Fig. 5b). Additional deletion after aa 2387 (Fig. 5b) disrupted the protein’s function. Those findings suggest that aa 1701–2387, though not required for NALCN binding, is essential for UNC80’s ability to potentiate *I*_NALCN_.

### UNC80 increases NALCN surface expression in a manner independent of UNC80’s distal C-terminal region

Our finding that the C-terminal 653 residues after aa 2554 appeared to be nonessential for *I*_NALCN_ was surprising (Fig. 5b). We and others previously reported that several patients with three truncational mutations within this C-terminal region had phenotypes including hypotonia, seizure, lack of speech development, and severe intellectual disability^42^ (Supplementary Fig. 1). Two such individuals carry c.2033del on one allele, leading to a premature truncation at Q678 (p.Q678Tfs*1, a likely null), and c.6657T>A on the other allele, leading to truncation at L2586 (p.L2586*, corresponding to L2654* in mouse UNC80). The C-terminal truncation mutation is in a region apparently not required for the whole-cell *I*_NALCN_.

**Fig. 6:**
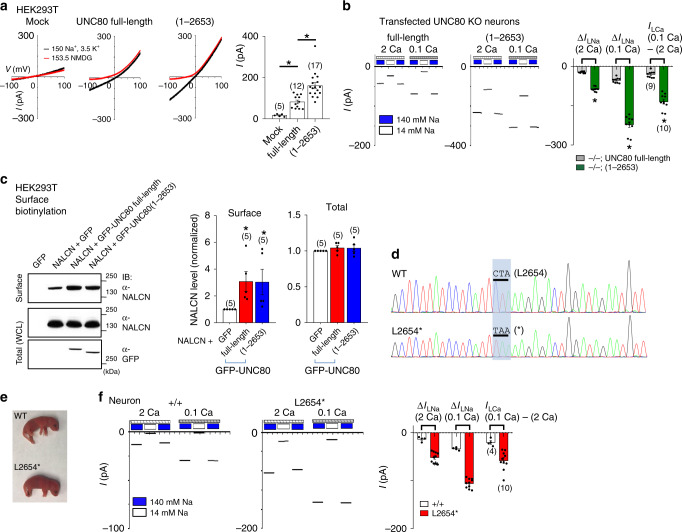
UNC80’s C-terminus truncated in human patients is not required for whole-cell *I*_NALCN_ but is essential for survival. **a**, **b** *I*_NALCN_ (**a** done as in Fig. 5b, averaged amplitudes at −100 mV given in the bar graph) or Na^+^-leak current (Δ*I*_LNa_) (**b**) from HEK293T cells (**a**) or cultured *UNC80* KO neurons (**b** recorded with 2 mM Ca^2+^ (2 Ca) or 0.1 mM Ca^2+^ (0.1 Ca) in the bath) transfected with full-length or mutant UNC80 truncated at L2654 (aa1–2653). **c** Surface biotinylation assays. Left, representative western blots. Surface proteins in cells transfected with cDNA combinations as indicated were biotinylated, isolated using streptavidin-conjugated beads, and probed with α-NALCN. Total protein (whole-cell lysate, WCL) was probed with α-NALCN or α-GFP. In the lower panel, GFP alone in the control lane migrated at ~20 kDa (outside the molecular range shown) and is not visible in the blot. Right, quantification. Signal intensity of each band from cells co-transfected with NALCN and full-length or truncated UNC80 was normalized to that from cells co-transfected with NALCN and GFP. **d** Sanger sequencing of the knock-in mice (L2654*, lower) and WT (upper). In the L2654* mice, the leucine-encoding codon (CTA, L2654) is mutated to a stop codon (TAA,*). **e** Representative appearances of WT and homozygous L2654* mutant P0 pups. For apnea phenotypes, see Supplementary Movie 2. **f** Δ*I*_LNa_ recorded from neurons cultured from WT and L2654* pups. Data are presented as mean values ± SEM. In the bar graphs, numbers of recordings or repeats are in parentheses. Two sample *t* tests were performed. Asterisk “*” indicates *p* < 0.05. *p* values are as follows. **a** (against the “full-length” group): “Mock” (*p* ≤ 0.001), “1–2653” (*p* ≤ 0.001), **b** (between the group of “−/−; UNC80 full-length” (full-length UNC80-transfected) and the group of “−/−; (1–2653)” (truncation mutant-transfected)): Δ*I*_LNa_ (2 Ca) (*p* ≤ 0.001), Δ*I*_LNa_ (0.1 Ca) (*p* ≤ 0.001), Δ*I*_LCa_ (0.1 Ca–2 Ca) (*p* ≤ 0.001), **c** (against the group of “GFP” (GFP-transfected)): “full-length” (*p* = 0.025), “1–2653” (*p* = 0.049). Source data are provided as a Source Data file.

As expected, UNC80 truncated at L2654 (containing the first 2653 residues, 1–2653, Fig. 6a) potentiated *I*_NALCN_ when transfected into HEK293T cells. It also fully rescued Δ*I*_LNa_ in *UNC80* KO neurons (Fig. 6b). The currents generated from the truncation mutant were significantly larger than those generated with full-length UNC80 in both HEK293T cells and in *UNC80* KO neurons. Like in WT, Δ*I*_LNa_ generated by the truncation mutant was also potentiated by lowering [Ca^2+^]_e_ (Fig. 6b).

Na_V_ and Ca_V_ β subunits potentiate channel currents partly by increasing the surface localization of the pore-forming α subunits (for reviews)^54,55^. We used the surface biotinylation assay to test whether UNC80 facilitates NALCN surface localization and whether the C-terminally truncated UNC80 is also able to do so. When co-transfected with NALCN in HEK293T cells, both the full-length and the UNC80 truncated at L2654 increased the surface fraction of NALCN by approximately threefold, without affecting the total levels of NALCN protein (Fig. 6c). Thus, the C-terminally truncated UNC80 is as efficient as the full-length one in increasing NALCN surface localization.

### UNC80’s distal C-terminal region is required for survival

A potential explanation for the apparent inconsistence between our findings of seemingly normal in vitro function of the C-terminally truncated UNC80 and the severe phenotypes in the human individuals is that the phenotypes found in humans are not directly caused by the *UNC80* mutation but are from other genetic variations yet to be discovered. To test this possibility, we generated a mouse line in which the codon (CTA) encoding L2654 was substituted with a stop codon (TAA) (Fig. 6d). Homozygous mutants (thereafter referred to as L2654*) were born close to a Mendelian ratio (9 litters, total of 76 pups, 24^−/−^, 22^+/+^, and 30^+/−^) (Fig. 6e). In neurons cultured from the mutant pups homozygous for L2654*, Δ*I*_LNa_ was significantly larger than that of WT, and like in WT, was increased by lowering [Ca^2+^]_e_ (Fig. 6f). Heterozygous mice were viable, fertile and did not have obvious abnormality. However, none of homozygotes survived beyond P1. Similar to the KOs, the newborn pups had severe apnea (Supplementary Video 2). The similar lethal phenotypes in the L2654* and in the null mice suggest that the C-terminal truncational mutations cause severe symptoms in humans.

### A C-terminal domain overcomes soma-retention for UNC80’s dendritic localization

**Fig. 7:**
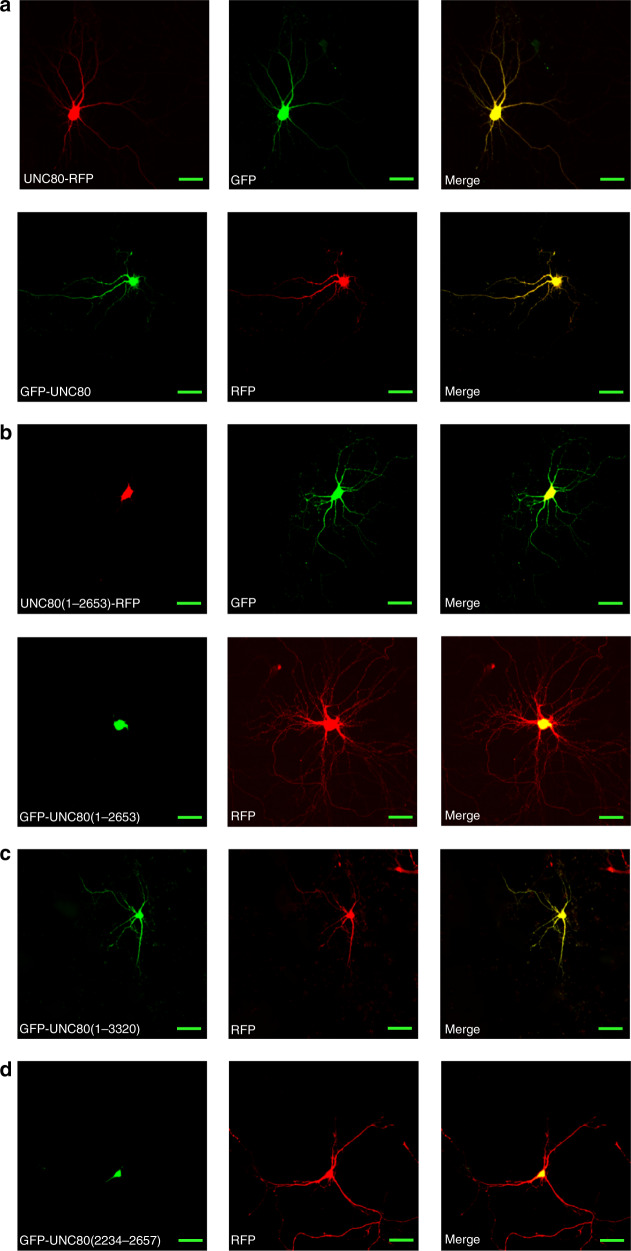
UNC80’s C-terminal domain overcomes soma-retention for dendritic localization. **a** C-terminally RFP (upper) or N-terminally GFP (lower)-tagged UNC80 was co-transfected with GFP (upper) or RFP (lower) into cultured wild-type hippocampal neurons. Scale bars: 50 μm. **b**–**d** Similar to (**a**) but transfected with UNC80 mutants truncated at L2654 (containing residues 1–2653) (**b**), lacking the last six residues (containing residues 1–3320) (**c**) or containing residues 2234–2758 only (**d**). Representative pictures from similar results of >10 are presented for each condition.

Another potential explanation of the severe phenotypes in mice and humans caused by the UNC80 C-terminal truncation could be that the mutant protein, though supporting functional currents, has aberrant localizations. When transfected into cultured hippocampal neurons, C-terminally RFP-tagged UNC80 (UNC80-RFP) was found in both soma and neuronal processes (Fig. 7a, upper panels). This ubiquitous localization was not an artifact of the RFP-tagging since N-terminally GFP-tagged UNC80 (GFP-UNC80) had a similar localization pattern (Fig. 7a, lower panels). In striking contrast, UNC80 truncated at L2654, whether with RFP-tagged at the C-terminus (Fig. 7b, upper panels) or with GFP tagged at the N-terminus (Fig. 7b, lower panels), was restricted in soma and was absent in axons and dendrites, suggesting essential roles of the C-terminus in the trafficking of UNC80.

We used additional truncations to narrow down the region required for UNC80’s dendritic localization. The last six residues (LDESHV; 3321–3326) of UNC80 contain a class I PDZ binding motif (S/T-X-Φ), and are highly conserved among vertebrates. Like WT, UNC80 lacking the last six residues were detected throughout neuronal processes (Fig. 7c). Similar localization patterns were also observed in UNC80 lacking the last 156 aa (UNC80 (1–3171)) (Supplementary Fig. 2a). Further deleting UNC80 from the C-terminus up to 326 aa (UNC80 (1–3000), Supplementary Fig. 2b) started to compromise UNC80’s localization, as the truncated protein had limited dendritic localization in some neurons (Supplementary Fig. 2b, upper panels) but was restricted to soma in the other (Supplementary Fig. 2b, lower panels). Together, these data suggest that the distal C-terminal ~300 aa (before ~aa 3000), while not required for the generation of whole-cell *I*_NALCN_, mediates UNC80’s dendritic localization.

The requirement of a C-terminal domain for dendritic localization also suggests the existence of a soma-retention signal that prevents UNC80’s diffusion from soma to dendrites. Indeed, UNC80 truncated at aa 2233 (Supplementary Fig. 2c), aa 1265 (Supplementary Fig. 2d), or aa 2387 (Supplementary Fig. 2e), similar to WT, was found in both soma and dendrites, suggesting the existence of the soma-retention domain between ~aa 2300–2600. Consistent with this idea, a fragment of 525 amino acids alone in the region (aa 2234–2657) was sufficient to retain GFP in soma (Fig. 7d).

### UNC80’s C-terminus interacts with UNC79

**Fig. 8:**
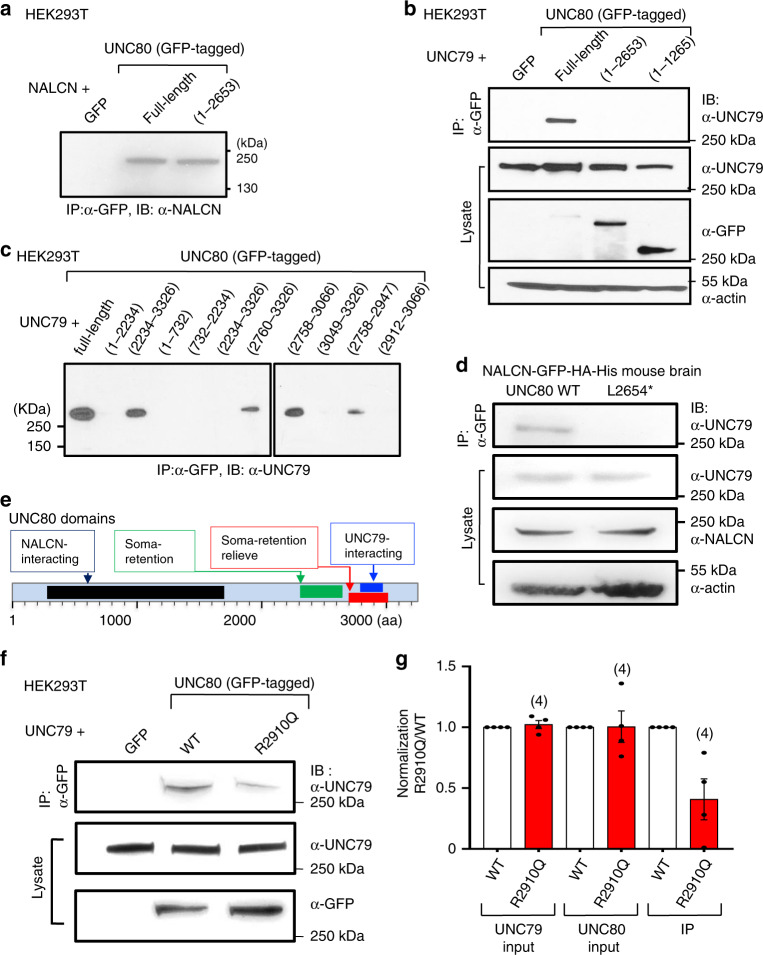
UNC80’s C-terminus contains an UNC79-interacting domain. **a** Association between UNC80 mutant truncated at L2654 (containing residues 1–2653) and NALCN. Cell lysates from HEK293T cells co-transfected with NALCN and GFP (as control), GFP-tagged full-length UNC80 or the truncation mutant were immunoprecipitated (IP) with anti-GFP and blotted with anti-NALCN. **b** Lack of association between UNC80 mutant truncated at L2654 and UNC79. Cell lysates from HEK293T cells co-transfected with UNC79 and GFP (as control), GFP-tagged full-length UNC80, or truncated UNC80 containing only residues 1–2653 or 1–1266 were immunoprecipitated (IP) with anti-GFP and blotted with anti-UNC79 (upper). Lower 3 panels: whole lysates were blotted with anti-UNC79, anti-GFP or anti-actin (for loading control). **c** Mapping UNC80’s UNC79-interacting domain. Cell lysates from HEK293T cells co-transfected with UNC79 and GFP-tagged full-length UNC80, or truncated UNC80 containing residues as indicated were immunoprecipitated (IP) with anti-GFP and blotted with anti-UNC79. **d** Disruption of UNC79-NALCN association in mouse brains lacking the C-terminal part of UNC80. Brain proteins were prepared from the NALCN-GFP-HA mice and those that also carry the homozygous L2654* mutation in the NALCN-GFP-HA-His background. Upper panel: NALCN was pulled down with anti-GFP antibody and the complex was probed with anti-UNC79. Lower 3 panels: whole lysates were blotted with anti-UNC79, anti-NALCN or anti-actin (lower 3 panels). **e** Summary of UNC80’s functional domains: NALCN-interacting domain (residues 301–1700), soma-retention domain (2387–2657), soma-retention relieve domain (2758-3000), and UNC79-interacting domain (2758–2947). **f**, **g** Reduced UNC80–UNC79 interaction strength associated with intellectual disability. **f** UNC79 was co-transfected with GFP, GFP-tagged wild-type UNC80 or GFP-tagged R2910Q UNC80 mutant in HEK293T cells. Immunoprecipitates (IP with anti-GFP, upper panel) and whole-cell lysates (lower two panels) were blotted (IB) with anti-UNC79 or anti-GFP as indicated. In the lower panel, GFP alone in the control lane migrated at ~20 kDa (outside the molecular range shown) and is not visible in the blot. **g** Protein levels normalized to that obtained with WT UNC80. Results were from four independent experiments. In (**a**–**d**, **f**), three or more independent repeats were performed with similar results. Data are presented as mean values ± SEM. Numbers of repeats are in parentheses. Source data are provided as a Source Data file.

UNC80 interacts with both NALCN and UNC79^20^. We used immunoprecipitation assays in transfected HEK293T cells to test whether the disease-associated L2654* truncation affects UNC80’s association with NALCN and UNC79. As expected from its ability to potentiate *I*_NALCN_, the truncated UNC80 was fully able to associate with NALCN (Fig. 8a).

In contrast to the WT, however, UNC80 lacking the last 554 aa failed to associate with UNC79 (Fig. 8b). Similarly, R1266* (corresponding to human R1265* found in patients^43^) did not associate with UNC79 (Fig. 8b). Additional truncation mutants located UNC80’s UNC79-association domain within a small region of 190 aa (aa 2758–2947, Fig. 8c). We also tested the inter-subunit association in the L2654* mouse brains. Consistent with the detection of *I*_NALCN_ in the mutant neurons, NALCN protein was present in the mutant brains (Fig. 8d). Unlike in the *UNC80* KO brains where UNC79 was undetectable (Fig. 1c), the level of UNC79 protein in the L2654* brain was similar to that of WT (Fig. 8d), suggesting that the physical interaction between UNC79 and UNC80 mediated by the fragment of aa 2758–2947 is not required for the UNC79 stability.

Based on co-immunoprecipitation experiments, we previously proposed that UNC80 bridges UNC79 into the NALCN complex^20,21^. We tested this model in brains using the L2586* mutant where both NALCN and UNC79 are present but the UNC79–UNC80 association is eliminated. Unlike in the WT, the association of UNC79 to the NALCN complex was absent in the L2654* mutant (Fig. 8d). These data suggest that the symptoms in the patients with truncations at the C-terminus (Supplementary Fig. 1) likely result from the inability of UNC80 to recruit UNC79 into the NALCN complex, in addition to mislocalization of UNC80.

### Reduced UNC80–UNC79 interaction strength is associated with intellectual disability

The L2654* mutation leads to neonatal lethality in mice and the corresponding mutation is associated with severe phenotypes in human^42^. Since the truncation eliminates the UNC80–UNC79 interaction domain (aa 2758–2947) and a highly conserved region in the C-terminal end (aa 2948–3207) outside the domain required for dendritic localization, we searched for more subtle mutations limited to the UNC79–UNC80 interaction to test specifically the function of UNC80–UNC79 interaction. A recently reported individual has a homozygous variation leading to an R2842Q substitution (corresponding to R2910Q in mice)^45^. R2910 is in the UNC79-interacting domain (aa 2758–2947, Fig. 8c, e) and is highly conserved in vertebrates. The individual can walk independently, has basic communication skills, and her symptoms are much milder than the ones with null UNC80 mutations or with the L2586* mutation. However, she is nonverbal and has severe intellectual disability^45^. To test whether R2910Q compromises UNC80–UNC79 interaction, we compared the ability of WT and R2910Q-bearing UNC80 to pull-down UNC79 in a co-immunoprecipitation assay. The mutant protein expressed at levels comparable to that of the WT. However, the apparent UNC80–UNC79 affinity was reduced by ~60% in the mutant (Fig. 8f, g).

## Discussion

In this study, we established the subunit composition of the NALCN complex and revealed UNC80’s functional domains important for *I*_NALCN_ potentiation, NALCN association, UNC79 association, soma-retention, and dendritic localization. Using KO mice and functional analysis, we also established a causal relationship between various UNC80 mutations and severe phenotypes in humans. In particular, UNC80 mutants truncated at the C-terminal end retain the protein’s somatic function, but lack dendritic localization and cause severe intellectual disability, suggesting the importance for the proper regulation of dendritic resting membrane potential through the NALCN channel.

There are now biochemical, functional, and genetic evidences that overwhelmingly support the idea that the Na^+^-leak channel has at least three subunits: NALCN, UNC80, and UNC79. First, essentially all the UNC80 and UNC79 protein is complexed with NALCN, as depleting NALCN from brain lysates also depletes UNC80 and UNC79. Second, UNC80 is required for the normal sizes and the regulation of *I*_NALCN_. Third, UNC80 and UNC79 control each other’s protein level. Finally, mutations in any of the three genes have comparable, although nonidentical, phenotypes. These include abnormal locomotion in *C. elegans* and *Drosophila*, severe apnea and neonatal lethality in mice, hypotonia and severe intellectual disability in humans^22–25,27,28,42–44^.

Several functional domains of UNC80 are now defined: an UNC79-interacting domain (U7-ID) in the C-terminal half, a NALCN association-domain (NALCN-ID) in the N-terminal half, a soma-retention domain, and a domain required to overcome the soma retention (Fig. 8e). In addition, the residues between 1700 and 2500, though not required for NALCN-association, are essential for *I*_NALCN_ potentiation (Fig. 5b). While the U7-ID is only 190 aa (aa 2758–2947), the NALCN-ID is as large as ~1500 aa and further deletion abolishes the UNC80–NALCN interaction. The requirement of such a large segment for UNC80–NALCN association suggests existence of multiple contacts between the two subunits in the tertiary structure. Further studies with structural analysis are required to reveal the details of the interaction.

UNC80 enhances *I*_NALCN_ likely through two major mechanisms. First, it increases the number of NALCN molecules on cell surface by controlling channel trafficking/insertion (Fig. 6c). This mechanism alone, however, is unlikely sufficient to explain the potentiation of *I*_NALCN_ by UNC80. UNC80 increases NALCN surface expression by approximately threefold when tested in HEK293T cells (Fig. 6c). In the UNC80 KO, which has a total NALCN protein level comparable to that of WT, *I*_NALCN_ is reduced by >> threefold, especially when recorded at 0.1 mM [Ca^2+^] (Fig. 2). In addition, both the full-length and C-terminally truncated UNC80 increase NALCN surface expression to comparable levels. *I*_NALCN_ with the truncated UNC80, however, is significantly larger than that with the full-length UNC80, both in transfected HEK293T cells and in neurons (Figs. 5, 6). The second mechanism by which UNC80 potentiates *I*_NALCN_ can be through the increase of channel opening via protein modification or inter-subunit interaction. UNC80 is known to mediate increases in *I*_NALCN_ by a Src-kinase pathway that can be activated by neuropeptides^15,16,18,19,56^. The resting level of *I*_NALCN_ presumably is influenced by the basal Src activity. The potentiation of NALCN by the auxiliary subunit UNC80 is reminiscent of that of Ca_V_ channels by the β subunits via the dual mechanism involving increase of surface expression level of the α1 subunits and the potentiation of channel opening^57,58^. Finally, overexpressing UNC80 can further increase *I*_NALCN_ above the level in WT (Fig. 2f), suggesting that UNC80 availability is a limiting factor in *I*_NALCN_. Regulating the protein level or modification of UNC80 could be another way to regulate the basal excitability of neurons.

The NALCN-mediated Na^+^ conductance is extremely small (~0.1 nS whole cell), generating only ~10 pA inward current in the hippocampal neurons at rest, almost at the detection limit of whole-cell patch clamp. This conductance is ~100- and 10-fold smaller than the peak Na_V_ and Ca_V_ conductances, respectively, making its contribution to the total conductance negligible during action potentials. However, at subthreshold MPs of approximately −70 mV, at which Na_V_s and HCNs are minimally open, NALCN is likely a major contributor for Na^+^ permeability.

Neurons lacking NALCN, though hyperpolarized toward *E*_K_ by ~10–20 mV, are at least partially functional and are able to generate action potentials^12,14–16^. Indeed, human patients with null NALCN or UNC80 mutations have normal muscle stretch reflexes, can raise hands above midline, and are able to perceive sounds, which require the basic functions of the nervous system to generate and transmit action potentials. It is perhaps surprising that the nervous system with neurons hyperpolarized by >10 mV still maintains some of the complex functions. However, the individuals lack eye fixation, normal communication and speech synthesis, and have some of the most severe intellectual disability. NALCN perhaps contributes to the complexity of the nervous system for higher cognitive function by helping generate and regulate RMP heterogeneity.

It’s known that in mammalian neurons the dendritic MPs can be significantly different from those of soma, and can fluctuate during behavior, as shown in brain slices and in freely behaving animals^59–62^. Voltage-gated ion channels such as K_V_s, Ca_V_s_,_ Na_v_s, and HCNs have been discovered in dendrites^63–65^ for reviews). Those channels regulate dendritic excitability and information integration. When transfected into cultured hippocampal neurons, NALCN, UNC80, and UNC79 are detected in soma, axons and dendrites. Similar wide-spread localizations of the complex were also observed in *Drosophila* and *C. elegans*^22–24,26^. Recording *I*_NALCN_ from dendrites is technically very challenging because of the small sizes of the current. Given the dominant function of UNC80 in controlling *I*_NALCN_, the localization of UNC80 likely regulates dendritic excitability. Like in soma, NALCN in dendrites perhaps primarily functions at MPs between the active ranges of HCNs and Na_V_s. Because of its small conductance, the channel regulates *P*_Na_/*P*_K_ and RMPs without significantly affecting the total dendritic impedance. Direct dendritic recordings from WT and mutant brain slices will be required to reveal the relative contribution of the NALCN complex to dendritic RMPs.

The dendritic localization of UNC80 is not simply by diffusion, but rather requires overcoming a somatic retention signal localized between aa 2234 and 2657 by the C-terminal segment (~aa 2600–3000). The dendrite localization domain also overlaps with the UNC79-interacting domain. In the UNC79 KO, there is a drastic reduction of UNC80 protein, suggesting that UNC79 is required for UNC80 stability^20^. In the UNC80 KO, the UNC79 protein level is also reduced. Intriguingly, the ability of UNC80 to stabilize UNC79 does not appear to require the UNC79-interacting domain since UNC79 level is normal in mice lacking this domain (L2654*, Fig. 8d). We cannot rule out additional interactions between UNC80 and UNC79 that are not strong enough to stand the detergent-containing immunoprecipitation assays used in the study but is responsible for the stability of each other. How UNC80 is trafficked to dendrites and whether it requires the UNC80–UNC79 interaction require further studies. In neurons with UNC80 lacking the C-terminus required for dendritic localization, we detected robust whole-cell *I*_NALCN_. Mice without the C-terminus are neonatal lethal, which precludes further behavioral studies. The human patients with similar C-terminal UNC80 truncations have basic motor skills, but lack fine motor coordination, do not have speech development, and have severe intellectual disability. Those severe phenotypes support the in vivo significance of UNC80’s dendritic localization and the importance of the proper regulation of dendritic RMPs.

## Methods

### Animals

Animal uses were approved by the University of Pennsylvania IACUC. Mice were housed under a 12 h light/dark cycle in rooms with ambient temperature of 19–26 °C and 30–70% humidity. KO and KI mouse lines were generated using the CRISPR/Cas9 technique^66^. Single-guide RNAs (sgRNAs) and Cas9 RNA were synthesized using in vitro transcription with MEGAshortscript T7 kit (for sgRNAs) or mMESSAGE mMACHINE T7 ULTRA kit (for Cas9 RNA), and purified using the MEGAclear kit (Life Technologies). The sgRNA-targeted sequences used for the generation of each line were (with PAM sequences in italic): TGAGTTCTATAATTATCTTT [*TGG*] and TCATCGTTGCCATTTATAAAA [*GGG*] (for *UNC80* knockout), TATCCAGTCTCTTTAGAATG [*AGG*] (for UNC80 L2654* KI), and TGACCTCCTGGATATTTAGA [*TGG*] (for GFP-HA-His triple-tagged *NALCN* KI). For the NALCN GFP-HA-His KI, the DNA donor contained 1.5 kb genomic DNA (left arm), followed by ~0.8 kb sequence encoding HA and 6 × His-tagged GFP at the C-terminus (GFP-HA-His) to replace the *NALCN* stop codon, and 6 kb genomic DNA (right arm). For the L2654* KI, single strand DNA (sequence:CATCATGGAGATGCTCCCCATTACTGATT

GGTCAGCAGAGGCTGTGAGGCCAGCTCTTATCCTCATT TAA AGAGACTGGA

TAGAATGTTCAACAAAATCCATAAGATGCCCACCTTGAGGTGAGAAGGC) was used as the donor. Cas9 RNA, sgRNA, and for KI, donor DNA were co-injected into embryos (done by the Transgenic and Chimeric Mouse Core of the University of Pennsylvania). Embryos used for injection were collected from C57BL6/J (JAX) (for UNC80 KO) or B6SJLF1/J (heterozygous for C57BL/6J and SJL/J, for L2654*, and *NALCN* KI). F0 founders were crossed to C57BL6/J to obtain germ-line transmission. Three independent *UNC80* KO sublines (deleted sequences indicated in Fig. 1a), three UNC80 L2654* KI sublines (sequence substitution indicated in Fig. 6d) and one NALCN GFP-HA-His KI line were established. Mice were backcrossed to C57BL6/J for two to ten generations before being used to generate P0 pups for neuronal culture. Littermates were used as controls. Genotyping was performed using PCR, restriction digestion, and/or Sanger sequencing. Sequences of PCR primers used for genotyping the UNC80 KO mice are TGATAACAAATGGGTTGCTATGTGAAGAAG (mU8.CRSP3.P0F, forward primer) and TGGAAATAAACAGTAAATCACAGACTAGG (mU8.CRSP3.P0R, reverse primer). Sequences of PCR primers used for genotyping the NALCN GFP-HA-His KI mice are AGATGACCTCCTGGATATTTAG (NCDWT.PF, NALCN-specific forward primer), GTGAACTTCAAGATCCGCCACAACATCG (EGFP.P3F, eGFP-specific forward primer), and TGAAAAACCCATGCTTGGGTGG (NL.CRKI.gtP1R, NALCN-specific reverse primer).

### Cell culture

HEK293T was purchased from ATCC and was not retested in-house for mycoplasma contamination. Cells were maintained at 37 °C and 5% CO_2_ in DMEM (Gibco) medium supplemented with 1× penicillin–streptomycin (Invitrogen) and 10% Fetal Bovine Serum (Atlanta biologicals). Neuronal cultures were made from P0 pups. Genders of the pups were not determined. Hippocampi were dissociated and digested with papain (Worthington). Cultured neurons were plated on 12 mm poly-L-lysine coated coverslips. The starting medium composed of 80% DMEM (Lonza), 10% bovine calf serum (Hyclone), 10% Ham’s F-12 (Lonza), and 0.5× penicillin–streptomycin. Medium was changed the next day (DIV1) to Neurobasal A medium (Gibco) supplemented with 1× B-27 (Gibco), 1× penicillin–streptomycin, L-glutamate (25 µM), and 0.5 mM Glutamax-I (Gibco), and on DIV2, to Neurobasal A medium supplemented with 1× B-27, 1× penicillin–streptomycin and 0.5 mM Glutamax-I.

### Transfection

HEK293T cells were transfected using Polyjet^TM^ (Signa Gen) transfection reagent. Transfected cells were replated on 12-mm poly-L-lysine coated coverslips ~48 h after transfection. Neurons between DIV 5 and 7 were transfected using Lipofectamine LTX (Invitrogen).

### cDNA constructs

WT non-tagged NALCN (rat), UNC79 (mouse), and UNC80 (mouse) used for transfections were described in^20^. N-terminally GFP-tagged WT and mutant mouse UNC80 constructs were made in the vector peGFP-C1 cut with EcoRI and ApaI. C-terminally mCherry RFP-tagged UNC80 constructs were made in EcoRI and XhoI cut vector pcDNA3.1(+), with mCherry amplified from the vector pmCherry-C1. Mutations were introduced using PCR fragments and assembled with T4 ligase or with the Gibson Assembly kit (NEB), and were confirmed by restriction digestion and Sanger sequencing.

### Electrophysiology

All experiments were performed at room temperature. Recording were done 48–60 h after transfection. For HEK293 cells, pipette solution contained (in mM) 150 Cs, 120 Mes, 10 NaCl, 10 EGTA, 4 CaCl_2_, 0.3 Na_2_GTP, 2 Mg-ATP, 0.002 Src family kinase activator (Santa Cruz), and 10 HEPES (pH 7.4). Bath solutions contained (in mM) 150 NaCl, 3.5 KCl, 1 MgCl_2_, 20 glucose, 2 CaCl_2_, and 10 HEPES (pH 7.4). In the NMDG^+^ bath, Na^+^ and K^+^ were replaced with NMDG^+^. For patch clamp recording with neurons, pipette solution contained (in mM) 120 CsCl, 4 EGTA, 2 CaCl_2_, 2 MgCl_2_, 4 Mg-ATP, 0.3 Tris-GTP, 14 phosphocreatine (di-tris salt), and 10 HEPES (pH 7.4). The 140 mM Na-containing bath contained (in mM) 140 NaCl, 5 KCl, 2 (or 0.1) CaCl_2_, 1 MgCl_2_, 6 glucose, 2 CsCl, and 10 HEPES (pH 7.4). In the 14 mM Na-containing bath, tris-Cl was used to replace 126 mM NaCl. TTX (1 µM), APV (10 μM), bicuculline (20 μM), and CNQX (20 μM) were applied in the bath to block Na_v_ and synaptic currents. Sodium-leak current (Δ*I*_LNa_) was measured by subtracting the holding currents obtained with 14 mM Na-containing bath from that obtained with 140 mM Na-containing bath^12^. Low [Ca^2+^]-activated leak current (*I*_LCa_) was measured by subtracting Δ*I*_LNa_ recorded in high (2 mM) Ca^2+^ from that obtained in low (0.1 mM) Ca^2+^. Patch clamp recordings were amplified and filtered at 1 kHz using a MultiClamp 700B amplifier and digitized at 5 kHz with a Digidata 1400A digitizer, both controlled with Clampex 10.4 (Molecular Devices). Data were analyzed using Clampfit 10.4 (Molecular Devices), Excel (Microsoft), and Origin (Origin Laboratory).

### Immunoprecipitation, Cell surface biotinylation, and western blotting (WB)

The anti-NALCN (used at 1 μg ml^−1^ for WB), anti-UNC79 (used at 1 μg ml^−1^), and anti-UNC80 (used at 1 μg ml^−1^) polyclonal antibodies used in this study have been described^18,20^. Other antibodies were from Sigma-Aldrich (anti-actin, #A5441, used at 1:1000 dilution for WB), Invitrogen (anti-GFP, #A11120, used at 1:1000 dilution for WB), and Rockland (anti-His, #600–401–382, used at 1:1000 dilution for WB). For HEK293T cells, cells from a 35 mm dish were lysed for 30 min with 360 µl IP buffer containing (in mM) 50 Tris-HCl (pH 7.4), 150 NaCl, 1% NP-40, and 1 EDTA supplemented with protease inhibitor cocktail (PIC, Roche). The IP buffer (RIPA) used in Fig. 8 also contained 0.5% (w/v) deoxycholate and 0.1% (w/v) SDS. Lysate was centrifuged at 20,000 × *g* for 30 min 4 °C. The supernatant was mixed with 1 µg antibody and incubated at 4 °C for 2 h. Samples were mixed with buffer-equilibrated protein A-agarose at 4 °C for 2 h, and washed with binding buffer 3 times (5 min each). Proteins were eluted with 1× lithium dodecyl sulfate (LDS) sample buffer (Invitrogen, #NP0007) containing 100 mM DTT.

For cell surface biotinylation assay, transfected cells were washed twice with PBS and incubated with Sulfo-NHS-LC-Biotin (Thermo Fisher, #21335, 0.5 mg ml^−1^ in PBS) for 30 min at 4 °C. Cells were washed with 100 mM glycine in PBS (1 time) and were lysed with 400 µl IP buffer and rotated at 4 °C for 1 h. Cell lysates were centrifuged 20,000 × *g* for 30 min at 4 °C. Sixty microliters supernatant was saved for input. Samples were mixed with 100 µl NeutrAvidin agarose (Thermo Fisher, #29200, pre-equilibrated 50% slurry) overnight at 4 °C, and were washed 3 times (5 min each) with IP buffer. Proteins were eluted with 1× lithium dodecyl sulfate (LDS) sample buffer (Invitrogen) containing 100 mM DTT.

For total brain protein prep from P0 pups used for WB in Fig. 1c, brains were homogenized in IP buffer without detergent and spun for 10 min at 5000 × *g*. Supernatant was collected, added with 1% NP-40 and spun for 20 min at 20,000 × *g*. Protein concentration was measured using the BCA assay (Pierce). Hundred micrograms of total protein was loaded onto each lane. For protein preparation from P0 pups used for immunoprecipitation in Fig. 8d, whole brains were homogenized in IP buffer, solubilized for 30 min, and spun for 30 min at 20,000 × *g*. One mg total protein in 500 μl was precipitated with 1 µg antibody for 2 h. Samples were mixed with 60 μl buffer-equilibrated protein A-agarose at 4 °C for 2 h, and washed with binding buffer 3 times (5 min each). Proteins were eluted with 30 μl 1× LDS sample buffer with 100 mM DTT and used for WB analysis. For membrane fractionation experiments described in Fig. 4, frozen adult brains were homogenized with dunce in buffer containing 250 mM sucrose, 5 mM Tris (pH 7.4), and 1× PIC. Homogenate was spun for 10 min at 1000 × *g* followed by another spin for 10 min at 3220 × *g* for 20 min. Supernatant (used as total protein) was spun for 2 h at 200,000 × *g* at 4 °C. Pellet (microsomal fraction) was resuspended in the same volume as starting volume of homogenization buffer. Equal volume of total and each fraction was loaded for WB. For affinity depletion experiments (Fig. 4), adult brains from the GFP-HA-His NALCN KI mice were homogenized in binding buffer containing 300 mM NaCl, 20 mM HEPES (pH8.0), and 1× PIC (EDTA-free). Homogenate was spun at 1000 × *g* for 10 min. The supernatant was mixed with CHAPS (1% final) for 1 h at 4 °C and spun at 18,000 × *g* for 20 min followed by mixing with pre-equilibrated nickel beads (Qiagen, 1 ml beads per 10 ml) for 1 h. The mixture was spun at 200 × *g* for 3 min. The supernatant was referred as Ni-depleted. For additional antibody depletion, one ml of supernatant was mixed with 20 μl agarose-conjugated anti-GFP (MBL) and mixed for 2 h at 4 °C.

For WB, proteins were separated using NuPage 4–12% Bis-Tris gels (Invitrogen) with denaturing running buffer (MOPS SDS running buffer, Novex), and transferred onto polyvinylidene difluoride (PVDF) membranes for 2–3 h. Membranes were pre-blocked with 5% nonfat dry milk in PBS with 0.1% Tween-20 (PBST) and incubated with primary antibodies at 4 °C overnight or at room temperature for 2 h. Following washes (2 times, 5 min each), membranes were incubated with HRP-conjugated secondary antibody for 1 h at room temperature, followed by 5× washes (5 min each) and additional incubation with detection reagents (Super Signal West Pico ECL from Thermo Scientific, or Hi/Lo Digital-ECL WB detection kit from Kindle biosciences). Signals were detected using X-ray films or with a camera (Fujifilm corporation digital camera (X-A2)).

### Protein localization

Hippocampal neurons on polylysine coated coverslips were transfected for 48 h using Lipofectamine LTX (Thermo Fisher). Cells were washed three times with PBS and fixed with 4% paraformaldehyde (in PBS) for 20 min at room temperature, followed by 5× washes with PBS. Samples were mounted with Prolong^TM^ Diamond Antifade Mountant (Invitrogen). Images were taken using a Nikon Eclipse Ti inverted microscope with a 20× lens using a 543 nm laser (for RFP) or a 488 nm laser (for GFP) for excitation.

### Clinical studies and whole-exome sequencing

The studies were approved by Western Institutional Review Board (WIRB, Protocol #20120789, Genetic Studies of Patients and their Families with Diseases of Unknown Genetic Etiology). The patient described in Fig. 3 is a 9-year-old male with global developmental delay, epileptic encephalopathy, hypotonia, ataxia, and a muscle biopsy suggestive of mitochondrial dysfunction. He was born by cesarean section due to nuchal cord and heart rate deceleration after an uneventful pregnancy. During the first year of life, he had feeding difficulties and was diagnosed with failure to thrive and gastroesophageal reflux disease. Parents noted he was missing his milestones at 6–12 months. He smiled at 6 months, babbled at 1 year, and crawled and cruised at 3 years. Two MRIs during this time were normal and a muscle biopsy indicated mitochondrial disease. At 4 years of age a repeat muscle biopsy showed Complex I and III deficiency by Oxidative phosphorylation enzymology testing and a decreased Complex I through clear native in-gel OXPHOS enzyme activity testing. Light microscopy of skeletal muscle showed mild to moderate increase in myofiber size variation that appeared to be due to early Type II fiber atrophy and increased presence of lipids. Urine organic acid testing showed significant ketosis and his blood pyruvate levels were high. CSF testing showed a glucose level of 33 (normal 60–80). At 6 years of age he had his first seizure. An additional MRI was normal and an EEG showed bilateral central, centrotemporal and centroparietal spike-wave discharges, with a fairly dramatic activation during sleep. A continuous EEG at around 7 years showed independent epileptiform discharges over the left and right central region during wakefulness, which were occasionally continuous over the right hemisphere as well as continuous epileptiform activity over the right central region during sleep, consistent with electrical status epilepticus. At 9 years of age a physical assessment showed mild hypotonia and choreoathetoid movements. He had good strength in his muscles and was able to walk with support but used a stroller. He was friendly, alert, and interactive; nonverbal but vocalized. He used a communication device to indicate choices. Dysmorphic features included triangular facies, mild frontal bossing, simplified shape of his auricles, and camptodactyly of the 4th and 5th digits of the right and 5th digit of the left hand. Electromyography and nerve conduction velocity testing, CSF exam for neurotransmitters, echocardiogram, lysosomal enzymes, VLCFA, MePCR for PWS, MED12, 7-dehydrocholesterol, lactate, ammonia, CPK, chromosomal microarray, Fragile X, DM1 and DM2, were all negative.

The participating family provided written consent and was enrolled into the Center for Rare Childhood Disorders at the Translational Genomics Research Institute (TGen). The patient was under 7 years of age at the time of enrollment and verbal assent was not required according to Western Institutional Review Board (WIRB Protocol #20120789). Whole blood was collected from the proband and parents for DNA extraction using the QiaAmp blood kit. Genomic libraries were prepared with the Illumina’s Truseq DNA Sample Preparation Kit (Illumina, Inc., San Diego, CA, USA), following the manufacturer’s protocol. A final sequencing library was prepared using the TruSeq Exome Library Prep Kit v1 and protocol from Illumina, Inc. WES was performed on the trio using the Illumina HiSeq2000 sequencing platform. Filtered reads were aligned to the Human genome (Hg19/GRC37) using the Burrows-Wheeler transform (BWA v.0.7.5)^67^. PCR duplicates were removed using Picard v1.92^68^ and base quality recalibration, indel realignment and SNP and indel discovery were performed using the Genome Analysis Toolkit (GATK v2.5–2)^69^. Data were filtered against dbSNP137, 1000 Genomes, an in-house exome database, and then annotated with SnpEff 3.2a against Ensembl v66 to identify novel damaging mutations. An annotated variant file containing variants in three family members was filtered to include novel, private, or rare variants according to the Exome Aggregation Consortium (ExAC) database and the Genome Aggregation Database (gnomAD). Mode of inheritance and disease association, followed by detailed analyst assessment for genotype–phenotype correlation, disease mechanism, and literature review were performed. Variants predicted to be damaging by multiple tools—the Combined Annotation Dependent Depletion (CADD), ExAC’s probability of loss-of-function intolerance score and missense *z*-score (pLi, *z*-score), Genomic Evolutionary Rate Profiling (GERP), Polymorphism Phenotyping v2 (PolyPhen2) algorithms were considered as candidate genes responsible for the child’s phenotype.

The patient is compound heterozygous for variants in the *UNC80* gene. On the paternal allele, he inherited the c.3883G>C, p.Glu1295Gln variant. On the maternal allele he inherited the double nucleotide polymorphism *c.1020_1021delGCinsTT, p.Gln340_P341delinsHS. Both variants were confirmed by Sanger sequencing. In the gnomAD database, which presumably only includes individuals without severe phenotype, both of the variations are also present in multiple heterozygous carriers (E1295Q variant in 30 heterozygotes and Q340_P341delinsHS in 152 heterozygotes and 1 homozygote), making a definitive genetic diagnosis of the severe phenotype found in our patient challenging. However, curators of the gnomAD database note that some individuals with severe disease may still be included in the dataset, albeit at a frequency equivalent to or lower than that seen in the general population. Therefore, the one homozygote could be an affected individual that was still included in the dataset.

### Quantification and statistical analysis

Origin 8.0 software was used for all electrophysiology data analyses. Protein level analysis (Fig. 6c) was done with Image J (US National Institutes of Health). Experimental sample sizes were chosen based on previous experiments to reach statistical significance and were not predetermined using statistical methods. Some of the experiments, but not all, comparing two groups were done in a blind fashion. The experimentalist was blind to the genotype during data collection and data analysis. No data were excluded for analysis. Comparisons between two groups were made using two sample *t* test (two sided). No adjustments were made. Numeric data were represented as mean ± SEM.

### Reporting summary

Further information on research design is available in the Nature Research Reporting Summary linked to this article.

## Supplementary information

Supplementary Info

Supplementary Movie_Audio_Data 1

Supplementary Movie_Audio_Data 2

Reporting Summary

## Data Availability

All relevant data are available from the authors. The source data underlying Figs. 1b, c, 2f, 3c, d, 4a–d, 5a, b, 6a–c, f, 8a–d, f, g are provided as a Source Data file. Web links of the publicly available datasets used in the study are: Genome Analysis Toolkit (GATK v2.5–2, https://gatk.broadinstitute.org/hc/en-us), dbSNP137 (https://www.ncbi.nlm.nih.gov/snp/), 1000 Genomes (https://www.internationalgenome.org/data/), SnpEff 3.2a (http://snpeff.sourceforge.net/), Ensembl v66 (https://m.ensembl.org/index.html), ExAC (http://exac.broadinstitute.org/), gnomAD (https://gnomad.broadinstitute.org/), the Combined Annotation Dependent Depletion (CADD, https://cadd.gs.washington.edu/), Genomic Evolutionary Rate Profiling (GERP, http://mendel.stanford.edu/SidowLab/downloads/gerp/), and Polymorphism Phenotyping v2 (PolyPhen2, http://genetics.bwh.harvard.edu/pph2/). Source data are provided with this paper.

## Source data

Source data
