# Supplementary material for: Intellectual disability-associated UNC80 mutations reveal inter-subunit interaction and dendritic function of the NALCN channel complex
Source: Nat Commun. 2020 Jul 3;11:3351. doi: 10.1038/s41467-020-17105-8 (PMC7335163; doi:10.1038/s41467-020-17105-8)
Supplement: Supplementary file 1 — Supplementary Info [file 41467_2020_17105_MOESM1_ESM.pdf]

Supplementary Information

Wie, et al., Intellectual Disability-Associated UNC80 Mutations Reveal Inter-subunit Interaction and Dendritic Function of the NALCN Channel Complex

Supplementary Figures
Supplementary Figure 1
Supplementary Figure 2

Supplemental Videos
Supplementary video 1. Apnea phenotype in the UNC80 KO pups.
Supplementary video 2. Apnea phenotype in the UNC80 L2654* mutant pups.

UNC80 variations associated with severe intellectual disability

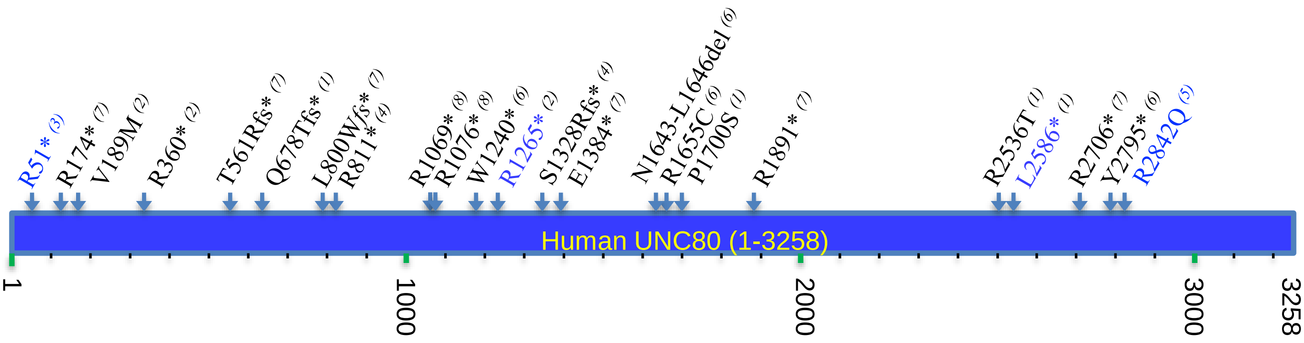

Supplementary Fig. 1. UNC80 variations associated with human diseases. Schematic presentation of mutations along the UNC80 open reading frame reported in human individuals with intellectual disability. Several mutations studied in this study are in blue (R51*, L2586* and R2842Q). See Fig. 8e for a summary of mapped functional domains. The access numbers for the UNC80 sequences are NM_032504 (human) and NM_175510 (mouse). References are (1) [1], (2) [2], (3) [3], (4) [4], (5) [5], (6) [6], (7) [7], (8) [8]

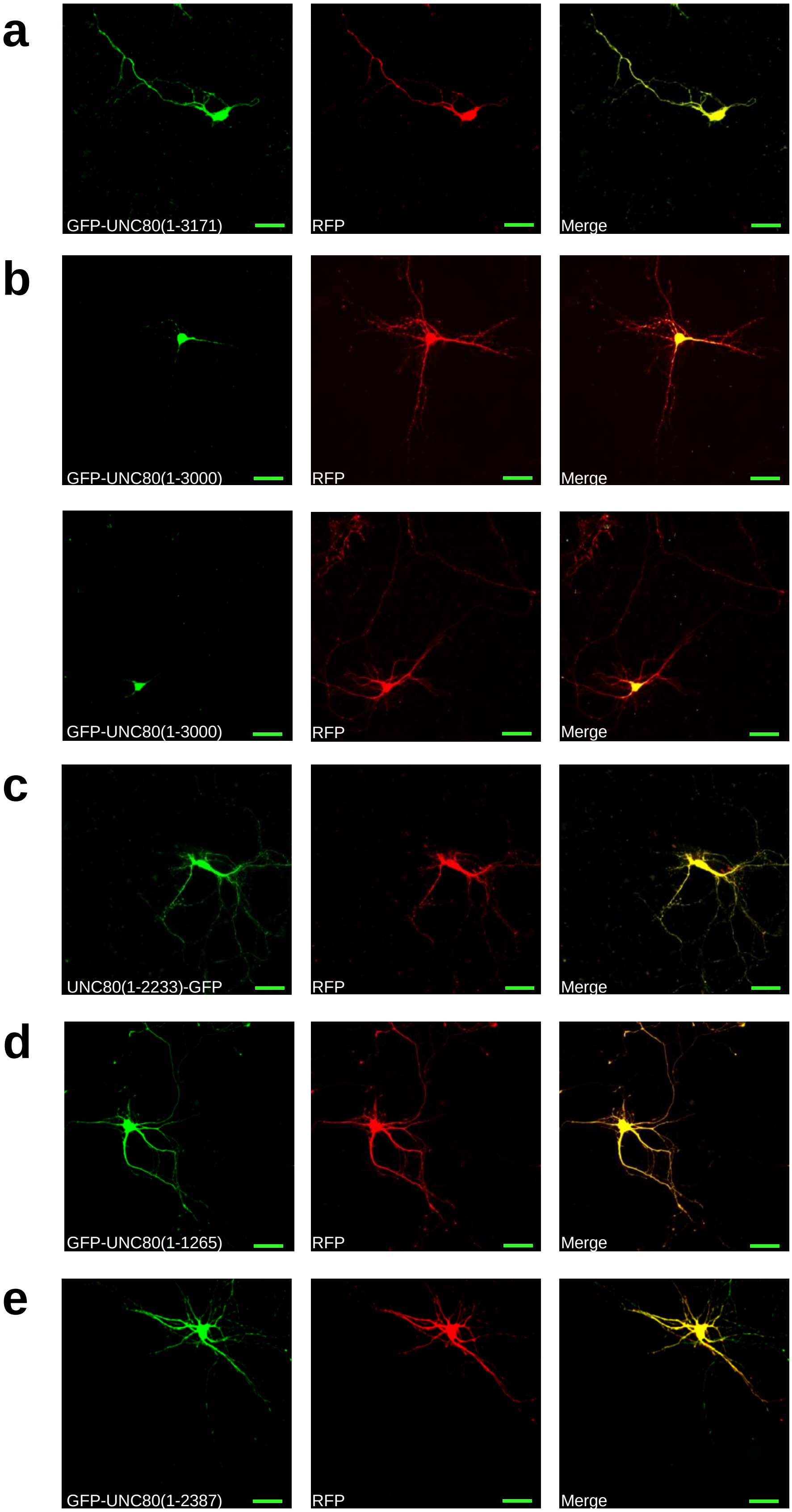

Supplementary Fig. 2. Localization of UNC80 C-terminal truncation mutants. GFP-tagged C-terminally truncated UNC80 containing residues 1-3171 (**a**), 1-3000 (**b**), 1-2233 (**c**), 1-1265 (**d**) or 1-2387 (**e**) was co-transfected with RFP (vector pmCherry-C1). In **b**, two representative neurons are shown for the 1-3000 truncation mutant, both with primarily soma localization, but one (*upper*) also with some dendritic localization. Representative pictures from similar results of >10 are presented for each condition. Scale bars: 50 μm.

References

1. Stray-Pedersen, A. *et al.* Biallelic Mutations in UNC80 Cause Persistent Hypotonia, Encephalopathy, Growth Retardation, and Severe Intellectual Disability. *American journal of human genetics* **98**, 202-209, doi:10.1016/j.ajhg.2015.11.004 (2016).
2. Shamseldin, H. E. *et al.* Mutations in UNC80, Encoding Part of the UNC79-UNC80-NALCN Channel Complex, Cause Autosomal-Recessive Severe Infantile Encephalopathy. *American journal of human genetics* **98**, 210-215, doi:10.1016/j.ajhg.2015.11.013 (2016).
3. Perez, Y. *et al.* UNC80 mutation causes a syndrome of hypotonia, severe intellectual disability, dyskinesia and dysmorphism, similar to that caused by mutations in its interacting cation channel NALCN. *Journal of medical genetics* **53**, 397-402, doi:10.1136/jmedgenet-2015-103352 (2016).
4. Valkanas, E. *et al.* Phenotypic evolution of UNC80 loss of function. *American journal of medical genetics. Part A* **170**, 3106-3114, doi:10.1002/ajmg.a.37929 (2016).
5. Obeid, T. *et al.* Identification of a novel homozygous UNC80 variant in a child with infantile hypotonia with psychomotor retardation and characteristic facies-2 (IHPRF2). *Metabolic brain disease*, doi:10.1007/s11011-018-0200-z (2018).
6. He, Y. *et al.* Biallelic UNC80 mutations caused infantile hypotonia with psychomotor retardation and characteristic facies 2 in two Chinese patients with variable phenotypes. *Gene* **660**, 13-17, doi:10.1016/j.gene.2018.03.063 (2018).
7. Bramswig, N. C. *et al.* Genetic variants in components of the NALCN-UNC80-UNC79 ion channel complex cause a broad clinical phenotype (NALCN channelopathies). *Human genetics* **137**, 753-768, doi:10.1007/s00439-018-1929-5 (2018).
8. Kuptanon, C. *et al.* Whole exome sequencing revealed mutations in FBXL4, UNC80, and ADK in Thai patients with severe intellectual disabilities. *Gene* **696**, 21-27, doi:10.1016/j.gene.2019.01.049 (2019).
