# Supplementary material for: Intellectual disability-associated UNC80 mutations reveal inter-subunit interaction and dendritic function of the NALCN channel complex
Source: Nat Commun. 2020 Jul 3;11:3351. doi: 10.1038/s41467-020-17105-8 (PMC7335163; doi:10.1038/s41467-020-17105-8)
Supplement: Supplementary file 4 — Reporting Summary [file 41467_2020_17105_MOESM4_ESM.pdf]

Corresponding author(s): Dejian Ren

Last updated by author(s): 6/9/2020

Reporting Summary

Nature Research wishes to improve the reproducibility of the work that we publish. This form provides structure for consistency and transparency in reporting. For further information on Nature Research policies, see our Editorial Policies and the Editorial Policy Checklist.

Statistics

For all statistical analyses, confirm that the following items are present in the figure legend, table legend, main text, or Methods section.

n/a	Confirmed	
☐	☒	The exact sample size (*n*) for each experimental group/condition, given as a discrete number and unit of measurement
☐	☒	A statement on whether measurements were taken from distinct samples or whether the same sample was measured repeatedly
☐	☒	The statistical test(s) used AND whether they are one- or two-sided<br>*Only common tests should be described solely by name; describe more complex techniques in the Methods section.*
☒	☐	A description of all covariates tested
☒	☐	A description of any assumptions or corrections, such as tests of normality and adjustment for multiple comparisons
☐	☒	A full description of the statistical parameters including central tendency (e.g. means) or other basic estimates (e.g. regression coefficient) AND variation (e.g. standard deviation) or associated estimates of uncertainty (e.g. confidence intervals)
☐	☒	For null hypothesis testing, the test statistic (e.g. F, t, r) with confidence intervals, effect sizes, degrees of freedom and P value noted<br>*Give P values as exact values whenever suitable.*
☒	☐	For Bayesian analysis, information on the choice of priors and Markov chain Monte Carlo settings
☒	☐	For hierarchical and complex designs, identification of the appropriate level for tests and full reporting of outcomes
☒	☐	Estimates of effect sizes (e.g. Cohen's d, Pearson's r), indicating how they were calculated

Our web collection on statistics for biologists contains articles on many of the points above.

Software and code

Policy information about availability of computer code

Data collection	there was no custom algorithm or software used.
Data analysis	there was no custom algorithm or software used.

For manuscripts utilizing custom algorithms or software that are central to the research but not yet described in published literature, software must be made available to editors and reviewers. We strongly encourage code deposition in a community repository (e.g. GitHub). See the Nature Research guidelines for submitting code & software for further information.

Data

Policy information about availability of data

All manuscripts must include a data availability statement. This statement should provide the following information, where applicable:

- Accession codes, unique identifiers, or web links for publicly available datasets
- A list of figures that have associated raw data
- A description of any restrictions on data availability

All relevant data are freely available from the authors. Figures 1-8 and Extended Data Figures 1 and 2 have raw data.

Field-specific reporting

Please select the one below that is the best fit for your research. If you are not sure, read the appropriate sections before making your selection.

☒ Life sciences ☐ Behavioural & social sciences ☐ Ecological, evolutionary & environmental sciences

For a reference copy of the document with all sections, see nature.com/documents/nr-reporting-summary-flat.pdf

Life sciences study design

All studies must disclose on these points even when the disclosure is negative.

Sample size	Sample sizes were not predetermined using statistical methods; they are chosen based on statistical analysis and standard in the field.
Data exclusions	No data were excluded from analyses.
Replication	All experiments were replicated and were consistent.
Randomization	Experiments were not randomized.
Blinding	Some experiments, but not all, comparing two or three groups were done in blind experiment which the investigators did not know the genotype or transfected DNA information until after data analysis

Reporting for specific materials, systems and methods

We require information from authors about some types of materials, experimental systems and methods used in many studies. Here, indicate whether each material, system or method listed is relevant to your study. If you are not sure if a list item applies to your research, read the appropriate section before selecting a response.

Materials & experimental systems

n/a	Involved in the study	
☐	☒	Antibodies
☐	☒	Eukaryotic cell lines
☒	☐	Palaeontology and archaeology
☐	☒	Animals and other organisms
☐	☒	Human research participants
☒	☐	Clinical data
☒	☐	Dual use research of concern

Methods

n/a	Involved in the study	
☒	☐	ChIP-seq
☒	☐	Flow cytometry
☒	☐	MRI-based neuroimaging

Antibodies

Antibodies used	Antibodies used were from Sigma-Aldrich (anti-actin, #A5441), Invitrogen (anti-GFP, #A11120) and Rockland (anti-His, #600-401-382). The anti-NALCN, anti-UNC79 and anti-UNC80 polyclonal antibodies used in this study were described in (Lu, B. et al. (2009) Nature 457, 741-744; Lu, B. et al. (2010) Neuron 68, 488-499). Antibody working concentrations and/or dilutions are given in the Methods section.
Validation	Antibodies were validated in-house using comparisons between samples from non-transfected and gene-specific transfected cells, and/or comparisons between wild-type and mutant tissues.

Eukaryotic cell lines

Policy information about cell lines

Cell line source(s)	HEK293T cell line was from ATCC.
Authentication	The cell line was authenticated by ATCC.
Mycoplasma contamination	HEK293T cells were tested by the provider.
Commonly misidentified lines (See ICLAC register)	none

Animals and other organisms

Policy information about studies involving animals; ARRIVE guidelines recommended for reporting animal research

Laboratory animals	Wild-type and mutant mice (BL6 background) were used, as described in section "Animals". Description of research mice used for each experiments can be found in the relevant figure legends.
Wild animals	No wild animal was used.
Field-collected samples	No field-collected samples were used.
Ethics oversight	The University of Pennsylvania IACUC.

Note that full information on the approval of the study protocol must also be provided in the manuscript.

Human research participants

Policy information about studies involving human research participants

Population characteristics	see page 21. One patient was enrolled for diagnosis and whole exome sequencing.
Recruitment	see page 21. One patient was enrolled for whole exome sequencing. The studies were approved by Western Institutional Review Board (WIRB, Protocol #20120789, Genetic Studies of Patients and their Families with Diseases of Unknown Genetic Etiology).
Ethics oversight	Western Institutional Review Board (WIRB)

Note that full information on the approval of the study protocol must also be provided in the manuscript.
